# Expression of Concern: Shikonin causes cell-cycle arrest and induces apoptosis by regulating the EGFR-NF-κB signalling pathway in human epidermoid carcinoma A431 cells

**DOI:** 10.1042/BSR-2015-0002_EOC

**Published:** 2023-01-19

**Keywords:** apoptosis, cell cycle, epidermal growth factor receptor–nuclear factor-kappa B signalling pathway, human epidermoid carcinoma cells, shikonin

The Editorial Office has been made aware of potential issues surrounding the scientific validity of this paper, hence has issued an expression of concern to notify readers whilst the Editorial Office investigates. Several images have been identified that seem to appear in other papers by unrelated authors. The Figure 3 dot plots seem to also appear in Figure 1E from Zhang et al. 2019 (https://doi.org/10.1186/s11658-019-0137-1) and Figure 4D from Liu et al. 2019 (https://doi.org/10.1186/s11658-018-0130-0). The Figure 2D GAPDH western blots appear (stretched and horizontally flipped) as the Figure 2A a-tubulin blots of Zeng et al. 2015 (doi: 10.3892/ijmm.2015.2292), and the Figure 2E GAPDH bands seem to appear as the Figure 4E B-actin bands of the same Zeng et al. 2015 paper. The Figure 5A B-actin bands also seem to appear as the Figure 6A GAPDH bands from Wang et al 2017 (doi: 10.1590/1414-431X20165714).

